# Lithium intoxication complicated by severe bradycardia after single-incision laparoscopic sleeve gastrectomy: a case report and literature review

**DOI:** 10.3389/fsurg.2025.1744520

**Published:** 2026-01-05

**Authors:** SiJun Xie, XiaoTian Pang, ZhengHang Yu, ZhongYang Zhang, Yuan Zhang, YiXing Ren

**Affiliations:** 1 Department of Gastroenterology Surgery, Affiliated Hospital of North Sichuan Medical College, Nanchong, China; 2 Institute of Hepatobiliary, Pancreatic and Gastroenterology, North Sichuan Medical College, Nanchong, China

**Keywords:** bipolar disorder, continuous renal replacement therapy, lithium toxicity, sinus bradycardia, sleeve gastrectomy

## Abstract

**Background:**

Lithium carbonate has a narrow therapeutic index, and postoperative anatomical/physiological changes after bariatric surgery may markedly alter its pharmacokinetics.

**Case:**

A 25-year-old woman with bipolar disorder on long-term lithium therapy developed altered consciousness and profound sinus bradycardia (nadir 27 bpm) approximately 7 weeks after single-incision laparoscopic sleeve gastrectomy. Laboratory testing revealed hyponatremia, acute kidney injury, and a serum lithium level of 4.16 mmol/L.

**Interventions and outcomes:**

Lithium and other psychotropics were discontinued, fluid resuscitation and inotropic support were initiated, and three consecutive sessions of continuous renal replacement therapy (CRRT) were performed. Serum lithium normalized without rebound, Sinus bradycardia recovered, and the patient was discharged without pacemaker implantation. During follow-up, lithium was permanently discontinued and replaced with lamotrigine. Mood remained stable without cardiac or neurologic sequelae.

**Conclusion:**

Post-bariatric patients receiving lithium should be considered high risk for intoxication. Routine monitoring with early recognition and multidisciplinary collaboration is essential to prevent complications. This case further shows that even extreme lithium-induced bradycardia can be fully reversible with timely withdrawal and extracorporeal clearance, highlighting the need to address reversible causes before permanent pacing.

## Introduction

With the increasing prevalence of bariatric surgery in the management of obesity and metabolic syndrome, medication management in patients with concomitant psychiatric disorders has attracted growing attention. Lithium carbonate, a first-line treatment for bipolar disorder, is characterized by a narrow therapeutic index and substantial toxicity risk. Its serum concentration is highly sensitive to changes in fluid status, renal function, and gastrointestinal absorption (1, 2). Postoperative anatomical and physiological alterations—including reduced gastric capacity, accelerated gastric emptying, decreased gastric acid secretion, insufficient fluid intake, and rapid weight loss—may disrupt lithium absorption, distribution, and clearance, thereby elevating the risk of toxic accumulation.

Current evidence is largely limited to case reports, which suggest that maintaining preoperative lithium dosages after sleeve gastrectomy (SG) or Roux-en-Y gastric bypass (RYGB) can lead to intoxication, sometimes accompanied by severe neuropsychiatric manifestations or cardiac arrhythmias (3, 4). However, systematic studies on the pharmacokinetic alterations of lithium after bariatric surgery and optimal dosing strategies remain scarce.

This report describes a young woman with stable psychiatric status and long-term regular lithium therapy who developed severe lithium intoxication approximately seven weeks after single-incision laparoscopic sleeve gastrectomy, presenting with marked sinus bradycardia and acute renal impairment. She achieved full recovery after lithium withdrawal, supportive care, and three CRRT sessions without rebound, and maintained psychiatric stability with significant weight loss during follow-up. To our knowledge, reports of post-bariatric lithium intoxication featuring extreme sinus bradycardia that fully reverses without pacemaker—along with sustained psychiatric stability—are scarce. Based on this case and a review of the literature, we discuss the potential mechanisms underlying lithium intoxication and cardiotoxicity after bariatric surgery, and propose monitoring and management recommendations for high-risk patients to inform clinical practice.

## Case presentation

### Overview of patient's medical history

A 25-year-old woman (160 cm, 83.2 kg, BMI 32.5 kg/m^2^) was scheduled for bariatric surgery due to obesity with metabolic syndrome. Her past medical history included non-alcoholic fatty liver disease, hyperlipidemia, hyperinsulinemia, thyroid dysfunction, hyperuricemia, and a 4-year history of bipolar disorder. The patient had a diagnosis of Bipolar I Disorder, with a history of two documented manic episodes and one major depressive episode prior to achieving stability on her current regimen. The psychiatric condition had remained stable for 2 years under maintenance therapy with lithium carbonate (600 mg twice daily), sodium valproate (500 mg twice daily), lurasidone (40 mg nightly), and propranolol (10 mg three times daily). Preoperative evaluation, including hematology, biochemistry, electrolytes, and upper gastrointestinal imaging, revealed no contraindications. Electrocardiography showed sinus tachycardia without structural abnormalities. All psychiatric medications were discontinued one day before surgery, and the final preoperative serum lithium concentration was 0.65 mmol/L. The patient underwent single-incision laparoscopic sleeve gastrectomy, with an uneventful intra- and perioperative course, and was discharged on postoperative day 3. Psychiatric medications were resumed thereafter, along with esomeprazole 40 mg once daily and bismuth potassium citrate granules 1.2 g three times daily for one month.

During the early postoperative phase, she developed intermittent postprandial vomiting (∼6 episodes/day), reduced appetite, and inadequate fluid intake (<2,000 mL/day). At 1 month, weight decreased to 70.6 kg (−12.6 kg). Lithium levels were not monitored. On POD 50, vomiting worsened with fatigue and chest discomfort. On POD 52, she presented to the emergency department with dyspnea, agitation, and abnormal behavior. Weight was 66.2 kg (a total reduction of 17.0 kg from baseline). Vital signs: blood pressure 120/61 mmHg, heart rate 39 bpm, respiratory rate 25 breaths/min, temperature 37.5 °C, SpO₂ 98%. Laboratory results: serum sodium 126 mmol/L (137–147), potassium 3.30 mmol/L (3.5–5.3), creatinine 286.7 μmol/L (41–73), urea nitrogen 9.23 mmol/L (2.6–7.5), and uric acid 619.1 μmol/L; estimated GFR 17.4 mL/min/1.73 m^2^. Cardiac biomarkers and pancreatic enzymes were within normal limits. Bedside ECG showed sinus bradycardia with T-wave abnormalities. Echocardiography revealed mild aortic/mitral/tricuspid regurgitation, mildly increased aortic valve velocity, and preserved biventricular function. Given suspected drug intoxication, electrolyte disturbance, and potential cardiac involvement, serum lithium was measured and returned at 4.16 mmol/L (therapeutic range 0.6–1.2 mmol/L), establishing acute lithium intoxication complicated by acute kidney injury and marked sinus bradycardia.

### Treatments and follow up

The patient's condition worsened, with confusion, lethargy, and shallow breathing. After multidisciplinary consultation, she was transferred to the ICU. Upon ICU admission, a formal psychiatric consultation was immediately obtained. The mental status examination during acute intoxication revealed marked impairment: the patient was disoriented to time and place, exhibited fluctuating consciousness with periods of agitation alternating with lethargy, and had slurred speech. She was unable to follow complex commands and demonstrated poor attention and memory recall. All psychiatric medications were immediately discontinued, and ECG monitoring was initiated. Heart rate fluctuated between 30 and 40 beats/min, with a minimum of 27 beats/min, respiratory rate 18 breaths/min, blood pressure 98/50 mmHg, and oxygen saturation 96%. Emergency treatment included continuous intravenous infusion of isoproterenol to enhance myocardial contractility and conduction velocity, intravenous atropine to increase heart rate, and fluid administration for volume expansion. Due to respiratory failure, the patient was intubated and assisted with mechanical ventilation (VC-AC mode; rate 12 breaths/min; VT 440 mL; PEEP 5 cmH₂O; FiO₂ 0.35). Bedside care maintained blood pressure at 110–130/67–87 mmHg, and heart rate 40–92 beats/min.

CRRT was initiated immediately after femoral vein catheterization. A bedside electrocardiogram (ECG) revealed sinus rhythm with supraventricular premature beats, while an echocardiogram revealed no new structural abnormalities. Given the patient's elevated lithium concentration and concurrent acute renal injury, three consecutive CRRT cycles were administered (Table 1), with the first two sessions delivered as continuous veno-venous hemodiafiltration (CVVHDF) and the third as continuous veno-venous hemodialysis (CVVHD). During treatment, the lithium concentration decreased from 4.16 mmol/L to 0.83 mmol/L, with no rebound observed during or after dialysis. As dialysis progressed, the patient's heart rate and blood pressure gradually recovered, and her consciousness improved. After the third CRRT cycle, an ECG revealed sinus rhythm with abnormal T waves. After discontinuation of isoproterenol, the heart rate remained at 92 beats/min, the respiratory rate at 16 breaths/min, and the blood pressure at 142/70 mmHg. Repeated liver and kidney function tests, electrolytes, and blood gas analysis gradually returned to normal. On the sixth day of hospitalization, the patient's blood lithium concentration dropped to 0.25 mmol/L, heart rate was 93 beats/min, respiratory rate was 22 breaths/min, blood pressure was 115/70 mmHg, and ventilation was attempted to be discontinued.

**Table 1:** Three cycles of CRRT process.

Session	Date and time	Mode	Vascular access	Anticoagulation	Duration (h)	Serum lithium (mmol/L)
1	2025-06-16 23:50–06-17 13:50	CVVHDF	Left femoral vein	Low molecular weight heparin	14	2.55
2	2025-06-17 18:00–06-18 07:30	CVVHDF	Right femoral vein	nafamostat + Low-dose Heparin	13.5	1.29
3	2025-06-18 15:00–06-19 02:00	CVVHD	Right femoral vein	regional citrate anticoagulation	11	0.83

CVVHDF, continuous veno-venous hemodiafiltration; CVVHD, continuous veno-venous hemodialysis.

On the evening of the eighth day after the initial extubation, the patient developed dyspnea and was reintubated. The primary causes were increased airway secretions and respiratory muscle fatigue. After a leak test on the 10th day of hospitalization, extubation was successfully repeated. A bedside ECG revealed sinus rhythm, a shortened PR interval, and abnormal T waves. Later in the hospitalization, the patient experienced occasional agitation, and the psychiatric department recommended the addition of quetiapine 100 mg nightly to help manage her mood. On the 15th day of hospitalization, an ECG showed no significant abnormalities, and her vital signs were stable, her consciousness was clear, and her liver and kidney functions had returned to normal. She was then discharged.

**Figure 1:**
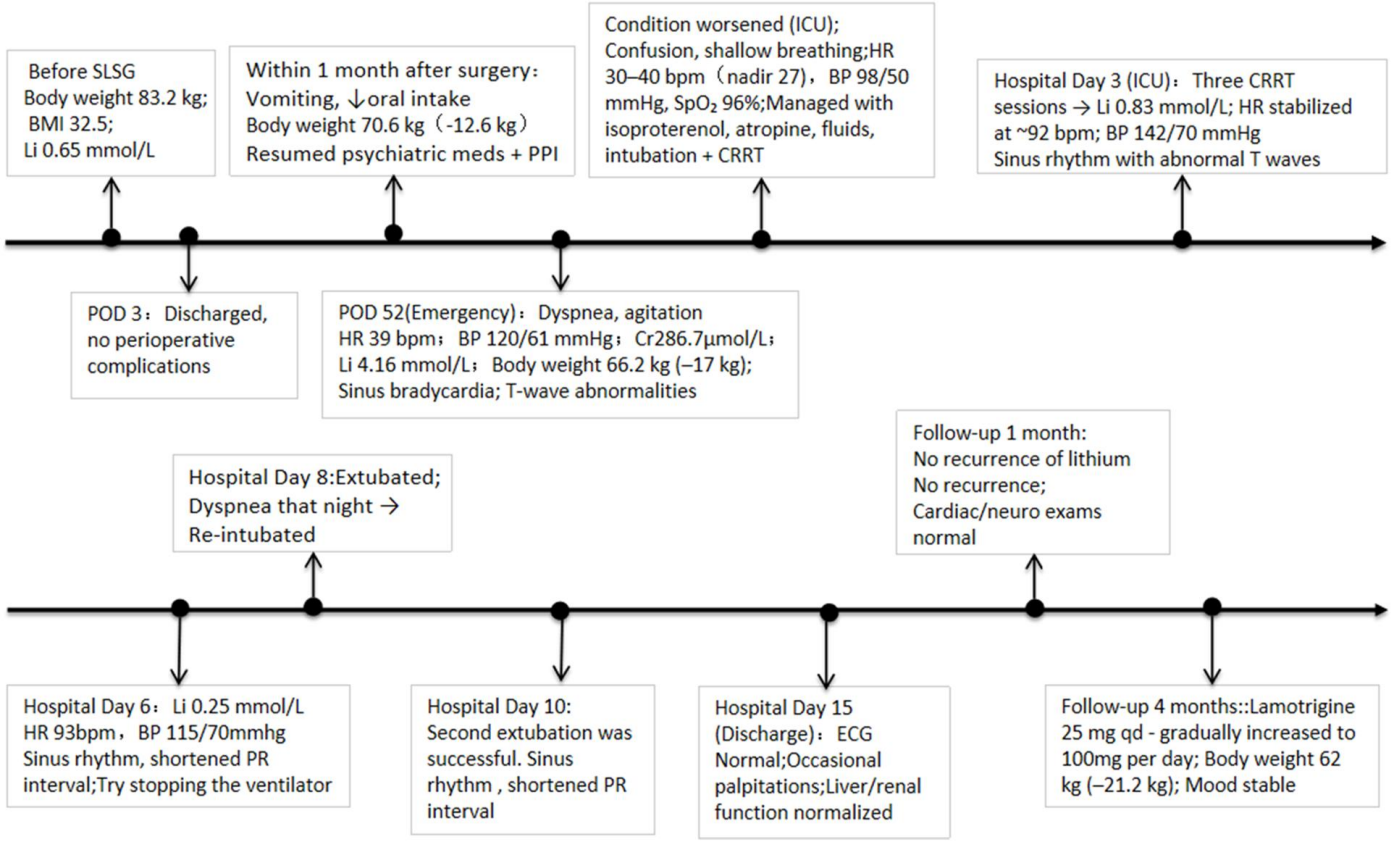
Timeline of clinical course after sleeve gastrectomy. SLSG, single-incision laparoscopic sleeve gastrectomy; POD, postoperative day; PPI, proton pump inhibitor; HR, heart rate; BP, blood pressure; ICU, intensive care unit; qd, quaque die; bpm, beats per minute; ECG, electrocardiogram.

After discharge, the patient discontinued lithium therapy. One month later, her mood remained stable, with no recurrence of lithium toxicity, complete renal function recovery, and normal cardiac and neurologic examinations. At follow-up four months after surgery, her weight had decreased by approximately 21.2 kg (to 62 kg). During outpatient psychiatric follow-up, lamotrigine was initially resumed at 25 mg once daily and was subsequently titrated to 100 mg/day over several weeks according to standard clinical practice. Valproate and lurasidone were not resumed post-discharge to avoid potential drug interactions and excessive sedation. Follow-up visits occurred approximately every 4–6 weeks, during which mood status was assessed through structured clinical interviews and standardized rating scales (e.g., YMRS and HDRS/PHQ-9), all of which remained within the non-clinical range. The patient reported good adherence and experienced no adverse reactions during titration. A chronological overview of the patient's clinical course, interventions, and outcomes is summarized in Figure 1.

## Discussion

To identify previously reported cases of lithium intoxication after bariatric surgery, we searched PubMed from database inception to September 2024 using the keywords “lithium”, “lithium toxicity” OR “lithium intoxication” combined with “bariatric surgery”, “sleeve gastrectomy”, or “Roux-en-Y gastric bypass”.This search showed that most reported patients developed symptoms within 1–2 months after surgery, typically presenting with neuropsychiatric manifestations (e.g., impaired consciousness, slurred speech, ataxia) or gastrointestinal symptoms (e.g., nausea, vomiting, diarrhea) (see Table 2). Arrhythmias are relatively uncommon, but when present they indicate more severe intoxication (3, 5). Among the few reported cases of bradycardia, Dahan et al. described a patient with a nadir heart rate of 36 beats/min after SG who ultimately required a permanent pacemaker because lithium could not be discontinued and sinus node dysfunction may occur even at therapeutic concentrations (3, 6). In contrast, Musfeldt et al. reported a case after RYGB with a minimum heart rate of 48 beats/min that recovered after drug withdrawal and fluid replacement, without pacing (5). However this case is distinguished by an exceptionally high serum lithium level (4.16 mmol/L) and an extremely low heart rate (27 bpm), yet sinus rhythm was completely restored without pacemaker implantation after lithium withdrawal and extracorporeal clearance.

**Table 2:** Reported cases of lithium intoxication after bariatric surgery.

Author/year	Age	Sex	SurgeryType	LithiumDose	OnsetTime	InitialSymptoms	SerumLithium (mmol/L)	Treatment	Psych Med after Discharge
Marques et al., 2020 (7)	44	F	RYGB	600 mg/day	POD 30	Confusion, muscle weakness, tremor, nausea, diarrhea	2.1	Stop lithium + hydration	Not specified
Jamison & Aheron, 2020 (8)	36	F	SG	300 mg AM + 600 mg PM	POD 180	Dizziness, nausea, fall, altered consciousness	1.63	Stop 2 days → reduce to 150 mg bid	Reduced to 150 mg bid, resumed 300 mg bid
Lin et al., 2020 (4)	39	M	SG	600 mg bid	POD 26	Watery diarrhea, dehydration, somnolence, altered consciousness	3.42	Stop lithium + 3 hemodialysis sessions	Switched to lamotrigine
Ayub et al., 2022 (9)	36	M	RYGB	300 mg tid	POD 38	Abdominal pain, nausea, vomiting, confusion, gait instability	2.2	Stop lithium + hydration	Reduced to 600 mg qd
Dahan et al., 2018 (3)	61	M	SG	900 mg/day	POD 60	General fatigue, tremor, bradycardia	1.6	Stop lithium + hydration + permanent pacemaker	Reduced to 600 mg qd
Musfeldt et al., 2016 (5)	61	F	RYGB	600 mg bid	POD 12	Dizziness, fatigue, hypotension, bradycardia	1.51	Stop lithium + fluids + vasopressors	Reduced to 300 mg bid
Alam et al., 2016 (10)	18	F	SG	300 mg AM + 600 mg PM	POD 35	Fatigue, diarrhea, tremor, confusion	2.7	Stop lithium + 2 hemodialysis sessions	Switched to olanzapine
Tripp, 2011 (11)	51	M	RYGB	450 mg AM + 600 mg PM	POD 14	Dehydration, altered consciousness	2.14	Stop lithium + hydration	Not mentioned
Dönmez et al., 2024 (12)	51	F	SG	1,200 mg/day	POD 37	Diarrhea, fatigue, confusion, tremor	2.85	Stop lithium + hydration	Switched to olanzapine
Present case	25	F	SLSG	600 mg bid	POD 53	Altered consciousness, severe bradycardia	4.16	Stop lithium + hydration + inotropes + 3 CRRT sessions	Switched to lamotrigine

SG, sleeve gastrectomy; RYGB, Roux-en-Y gastric bypass; POD, postoperative day; CRRT, continuous renal replacement therapy; F, female; M, male; SLSG, single-incision laparoscopic sleeve gastrectomy.

Current guidelines recommend permanent pacing only for symptomatic sinus node dysfunction that persists despite correction of reversible causes (13). Consistent with this principle, our case demonstrates that even profound bradycardia (27 bpm) associated with lithium intoxication can be completely reversible after drug withdrawal and extracorporeal clearance, without pacemaker implantation. Nevertheless, in patients unable to discontinue lithium or with intrinsic conduction system disease, pacing should be considered individually.

The mechanism of lithium-induced cardiotoxicity remains incompletely understood, but several pathways have been proposed: inhibition of the *β* receptor–adenylate cyclase pathway (negative chronotropic effect) (14); interference with the Na^+^/Ca^2^^+^ exchanger and Na^+^/K^+^ pump activity leading to conduction abnormalities (6, 15); and activation of ATP-sensitive potassium channels with inhibition of calcium channels, resulting in further conduction and contractile dysfunction (16). These mechanisms may together explain the extreme bradycardia and circulatory instability observed in our patient, though higher-level evidence is required.

After bariatric surgery, lithium intoxication typically results from converging risk factors, several of which were present in this patient. Long-term propranolol use for thyroid dysfunction exerts a negative chronotropic effect that could aggravate bradycardia in the setting of lithium toxicity. Esomeprazole, resumed postoperatively, suppresses gastric acid and raises intragastric pH, theoretically enhancing lithium absorption (10). Despite a daily dose of 1,200 mg sustained-release lithium carbonate, the patient also used other mood stabilizers; prior studies suggest that concomitant mood stabilizers, antiepileptics, or antipsychotics may potentiate lithium toxicity (17). This drug interaction mechanism caused by combined use has rarely been reported in previous literature, but it may be an important factor in aggravating poisoning in this case.

Rapid weight loss may lead to changes in lithium pharmacokinetics. The patient in this case had lost about 17 kg before the onset of symptoms. Previous studies studies suggest that obese individuals have higher lithium clearance preoperatively, while rapid weight reduction decreases glomerular filtration rate (GFR), total body water, and distribution volume (Vd), thereby predisposing to lithium accumulation under a fixed dose (18, 19). Intermittent vomiting and insufficient fluid intake (<2,000 mL/d) further reduced renal perfusion and caused a hyponatremia state. Due to the competitive transport of lithium and sodium in the proximal tubules, lithium reabsorption is also enhanced, further increasing serum lithium levels (3, 20). One month post-surgery, the patient crushed the lithium tablet and took it due to dysphagia. Studies have shown that crushed or liquid lithium releases more rapidly in the altered gastrointestinal environment, potentially significantly increasing absorption rate and peak concentration (21). Combined with the anatomical and physiological changes that occur after sleeve gastric surgery, such as reduced gastric capacity, accelerated gastric emptying, and decreased gastric acid secretion (10, 22), these factors together explain the very high serum lithium level and severe cardiotoxicity in this case.

In severe lithium poisoning, supportive care alone is insufficient. The EXTRIP Workgroup recommends extracorporeal removal when serum lithium exceeds 4.0 mmol/L or when neurological, cardiac, or renal complications occur (23). Intermittent hemodialysis (IHD) is first-line, but CRRT is acceptable for unstable patients (24). Given the patient's hypovolemia, hemodynamic instability, and marked sinus bradycardia, IHD could further worsen instability and arrhythmic risk; therefore, CRRT was selected to achieve continuous and gentle lithium removal and maintain circulatory stability. Lithium levels decreased from 4.16 to 0.83 mmol/L over three sessions, with no rebound observed. It should be pointed out that the redistribution of lithium between blood and tissues can lead to rebound after the cessation of extracorporeal treatment. This phenomenon is most pronounced after high-efficiency IHD but may also occur following CRRT, albeit typically to a lesser extent (25). Therefore, post-cessation retesting of serum lithium levels, with repeat sessions as needed, should be standard practice.

During the subsequent short-term follow-up, the patient's mood remained clinically stable after transitioning from lithium to a lamotrigine-based regimen. Lamotrigine was titrated from an initial 25 mg/day to a target dose of 100 mg/day, while valproate and lurasidone were discontinued. This stability was achieved in the context of a significant weight loss of 21.2 kg. Animal and observational studies suggest that obesity-related systemic inflammation and altered 5-HT signaling may contribute to mood dysregulation (26, 27), and that weight loss can mitigate inflammation and improve affective symptoms (28). Although direct evidence in bipolar disorder remains limited and largely observational, the substantial weight loss in this case may have synergized with lamotrigine to facilitate stability under a simplified medication regimen. These findings, however, are derived from a short-term observation, and prospective, long-term studies are needed to confirm the durability of this outcome and the precise role of weight loss in the long-term management of bipolar disorder post-bariatric surgery.

## Conclusion

Patients using lithium after bariatric surgery should be regarded as high-risk for intoxication, and routine postoperative monitoring with early recognition is essential. Multidisciplinary collaboration between psychiatry and metabolic surgery is critical to prevent severe complications. Importantly, this case also demonstrates that even extreme sinus bradycardia from lithium intoxication can be fully reversible with timely drug withdrawal and extracorporeal clearance, underscoring the need to address reversible causes before considering permanent pacing.

## Data Availability

The original contributions presented in the study are included in the article/Supplementary Material, further inquiries can be directed to the corresponding author.
